# Prevalence of pendrin defects in sudanese families with congenital hypothyroidism

**DOI:** 10.1007/s12020-025-04423-4

**Published:** 2025-09-16

**Authors:** Mohammad S. Islam, Alexandra M. Dumitrescu, Amna Ahmed, Samuel Refetoff, Roy E. Weiss

**Affiliations:** 1 Department of Medicine, University of Miami Miller School of Medicine, Miami, FL, USA; 2 Departments of Medicine, Committees on Molecular Medicine and Nutrition, The University of Chicago, Chicago, IL, USA; 3 Department of Paediatrics and Child Health, Faculty of Medicine, University of Khartoum, Khartoum, Sudan; 4 Departments of Medicine and Pediatrics, Committee on Genetics, The University of Chicago, Chicago, IL, USA

**Keywords:** Pendred syndrome, *SLC26A4*, Hearing loss, Goiter, Dyshormonogenesis

## Abstract

**Purpose:**

Pendred syndrome (PDS) is an autosomal recessive disease caused by variants in *SLC26A4* manifesting thyroid dyshormonogenesis. Patients typically present with goiter and sensorineural hearing loss (SNHL). The prevalence of PDS in non-African populations is estimated to be between 7.5 and 10 per 100,000, while its occurrence in African populations has not been reported with molecular analysis.

**Methods:**

This study, conducted at a university research center in Miami, USA and Khartoum, Sudan, to investigate PDS in Sudanese families with congenital hypothyroidism (CH). It involved 32 Sudanese families with children diagnosed with CH between 2016 and 2023. Patients underwent clinical evaluation, thyroid function tests, and genetic sequencing.

**Results:**

Two disease-causing *SLC26A4* variants were identified in two consanguineous families with first-cousin parents. One homozygous nonsense variant causing premature termination, p.Trp482*, previously reported as part of a compound heterozygous defect together with p.Gly102Arg, while the other homozygous defect was a previously reported missense variant, p.Thr410Met. In 32 families (72 individuals) whole exome sequencing data revealed 56.3% of families or 45.8% individuals harbored the *SLC26A4* variants either in hetero or homozygous state. Of the 33 subjects who tested positive for the variants, 12 (36.4%) harbored more than one *SLC26A4* variant.

**Conclusions:**

This report extends our understanding of the severity of the phenotypes caused by deleterious bi-allelic variants in *SLC26A4*. Recurrent *SLC26A4* variants observed in our cohort likely reflect high consanguinity rather than a founder effect. *SLC26A4* screening could be a part of the molecular testing for children presenting with congenital or early-onset SNHL in Sudan.

## Introduction

Dyshormonogenesis accounts for 15–20% of congenital hypothyroidism (CH) worldwide. The rate of dyshormonogenesis in the Sudanese population is 60% or 3 times that in the non-Sudanese population. High rates of consanguinity contribute to the abnormally high prevalence while low levels of iodine intake are accountable for the severity of the dyshormonogenesis. Among the many genes, Dual-oxidase 1 (*DUOX1*), Dual-oxidase 2 (*DUOX2*), Iodotyrosine deiodinase (*IYD*), several solute-carrier family member genes (SLC) including *SLC26A7* and *SLC5A5*, thyroid peroxidase (*TPO*), and thyroglobulin (*TG*) have been described the Sudanese population with CH [1].

Pendred syndrome (PDS, OMIM 274600) is an autosomal recessive disease characterized by sensorineural hearing loss (SNHL), congenital and severe to profound temporal bone abnormalities, goiter, and iodide organification defects [2–5]. Impaired hearing is the hallmark of PDS, which is associated with an enlarged vestibular aqueduct (EVA) due to inner ear malformations [6]. The hearing loss is typically bilateral, severe to profound, and prelingual. In some cases, progressive and/or post lingual hearing impairment is presented [7]. PDS is caused by homozygous or compound heterozygous variants in the *SLC26A4* (member 26 of the solute linked carrier family A4, OMIM 605646) gene, located on chromosome 7q31, which encodes pendrin, a multifunctional anion exchanger highly expressed in the thyroid and in the inner ear [2, 3, 8–10].

In the thyroid, pendrin is expressed at the apical membrane of thyroid cells [11]. The chloride-iodide exchanger role helps transport iodide from the cell to the colloid in the follicular lumen, where organification of iodide takes place [12]. Defective iodide organification occurs from the loss of *SLC26A4* function. Consequently goiter develops in most affected individuals and one third of subjects with PDS develop hypothyroidism [4]. In the inner ear, pendrin functions as a chloride/bicarbonate exchanger that is essential for maintenance of the endolymph homeostasis [13]. Pre- or perilingual development of deafness or milder forms of hearing loss are frequently associated with malformations of the inner ear called EVA and Mondini’s cochlea [10, 14].

The prevalence of PDS is estimated to be between 7.5 and 10 in 100,000 individuals [2, 15]. PDS variants with high heterogeneity and ethnical differences were reported previously [16]. A splice-site variant, c.919–2 A > G was reported to account for more than 80% of variants in Taiwan [17, 18] and another variant c.1001 + 1G > A was one of the 2 most frequent variants in the United States [19]. To date, approximately 150 variants have been identified in the *SLC26A4* gene of patients with PDS [20]. However, information on the incidence in African populations is limited. We present the first cases of *SLC26A4* defects and PDS in this population, providing important information on diagnosis and genetic counseling.

## Case presentations

**Fig. 1:**
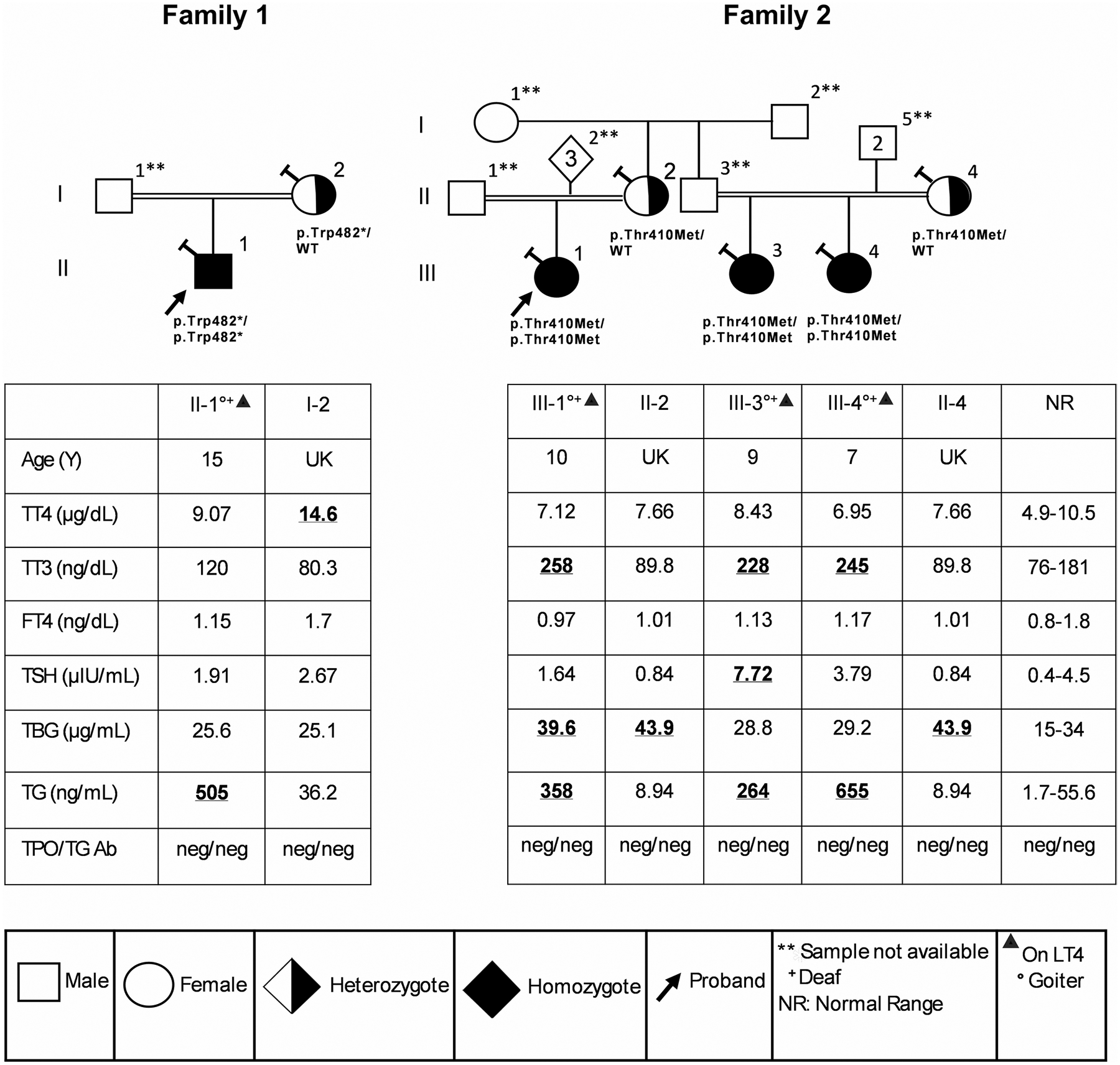
Pedigrees and thyroid function tests of 2 families with CH caused by*SLC26A4*variants. Family 1 (c.1446G > A, p.Trp482*) and Family 2 (c.1229 C > T, p.Thr410Met). Generations are denoted by roman numerals. Each subject is identified by the number just above the corresponding symbol. Results of thyroid function tests are aligned below each symbol. Diamond shape with inside number indicates the number of additional subjects of either sex. Abnormal values are in bold and underlined. The Asterisk (*) symbol indicates the stop codon. Slanted “T” on the upper left of the symbol indicates the individuals tested in Miami. A double horizontal line between individuals indicates consanguinity. Abbreviations: FT4, free thyroxine; TBG, thyroxine-binding globulin; TG, thyroglobulin; TG Ab, thyroglobulin antibody; TPO Ab, TPO antibody; TSH, thyroid-stimulating hormone; TT3, total triiodothyronine; TT4, total thyroxine; UK, unknown

Family 1: A 15-year-old East Sudanese boy (Fig. 1, Family 1: II-1) from asymptomatic consanguineous parents was found to have a goiter on routine examination at age 5. He was also diagnosed with bilateral SNHL and associated speech difficulties. There was no jaundice, constipation, umbilical hernia, or dysmorphic features. His breast feeding was satisfactory, and skin was not dry. Family history revealed a maternal cousin with hearing impairment, but the sample was not available for the study. Based on clinic notes available, the serum thyroid stimulating hormone (TSH) level was elevated in measurements performed in Sudan, and the patient was treated with 100 μg levothyroxine (L-T4) with normalization of the serum TSH. The mother had no goiter nor had hearing abnormalities. The father was unavailable for study.

Family 2: A 10-year-old Southeastern Sudanese girl (Fig. 1 Family 2: III-1) from consanguineous parents, presented at 2 years of age with speech delay, motor delay and small goiter. Evaluation at age 8 revealed moderate hearing loss in the left ear and moderate to severe loss in the right ear, associated speech difficulties, and a large goiter. She received 50 μg L-T4 for 3 months and her TSH and free thyroxine (FT4) levels were normal (prior thyroid function tests were not available). She did not follow up with the health care provider for 2 years. Family history revealed a goiter in her father. Blood samples were not available from her father and three siblings. However, maternal cousins (Central Sudan) (Fig. 1, Family 2: III-3, III-4) from asymptomatic consanguineous parents were available for the study. Individual III-3 presented with hearing loss and speech delay at age 2 and goiter at age 5. Maternal cousin III-4 (Fig. 1, Family 2) was also diagnosed with bilateral SNHL, speech difficulties and goiter. Subsequently, III-3 and III-4 were placed on 75 μg L-T4 due to elevated TSH. We obtained blood at analysis of thyroid function at the ages listed in Fig. 1. The maternal aunt’s sample (Family 2, II-4) was available for thyroid function and genetic studies. Of note one of two additional cousins was suspected at 3 years old of having hearing impairment during the last medical office visit. The mother declined hearing testing. Samples were not available for these additional two cousins.

## Materials and methods

Patients displaying stigmata of CH were specifically assessed by the pediatric endocrinologist at the University of Khartoum, Sudan, through a targeted selection rather than random sampling. Written informed consents were taken from the patients or their guardians and their participating family members. All research was conducted in conformity with the defined ethical standards of the declaration of Helsinki and was approved by the institutional review board (IRB) of University of Miami Miller School of Medicine. Thyroid tests, TSH and FT4 were done at the time of diagnosis in Sudan. Subsequent thyroid function tests (TFTs) were performed on Immulite^®^ 1000 (Siemens, Munich, Germany) platform in Miami.

Genomic DNA was extracted from the peripheral blood cells using the QIAmp^®^ DNA Blood Mini Kit (Qiagen, Germany) at the University of Miami. Whole exome sequencing (WES) was conducted using the IDT xGen Exome Hyb Panel v2 for library preparation, followed by sequencing on the Illumina NovaSeq X Plus platform with 2 × 150 bp paired-end reads, generating approximately 52 million reads per sample. The analysis was performed by Admera Health (South Plainfield, New Jersey). Sequencing achieved a mean target coverage of 53.6×, ensuring sufficient depth for variant calling. The bioinformatics pipeline included adapter trimming, read alignment to the Genome Reference Consortium Human Build 38 (GRCh38) reference genome using Burrows-Wheeler Aligner-Maximal Exact Matches 2 (BWA-MEM2), and variant calling through the Genome Analysis Toolkit (GATK) Best Practices workflow. Variants were annotated using public databases including Clinical Variants (ClinVar), Database of Single Nucleotide Polymorphisms (dbSNP), Genome Aggregation Database (gnomAD), Catalogue Of Somatic Mutations In Cancer (COSMIC), and Database for Nonsynonymous Functional Predictions (dbNSFP). Variants were filtered based on allele frequency, zygosity, predicted functional impact, and inheritance patterns. Quality control metrics included assessments of total reads, mean coverage, duplication rate, and percentage of target bases covered at ≥ 20×, all of which met standard thresholds for high-confidence variant detection. Sanger sequencing (Genewiz, Abi 3730xl DNA Analyzer) was used to confirm those variants in both forward and reverse directions. In silico tools like Sorting Intolerant From Tolerant (SIFT) [21], Polymorphism Phenotyping version 2 (PolyPhen2) [22], Mutation Taster [23], Functional Analysis Through Hidden Markov Models (FATH-MM) [24], Fast and efficient meta-analysis of genome wide association scans (METAL) [25], Mutation Assessor [26], Rare Exome Variant Ensemble Learner (REVEL) [27], and The American College of Medical Genetics and Genomics (ACMG) classification further evaluated the detrimental effects of the identified variations [28].

## Results

Thyroid function tests of the probands and available family members are reported on thyroid hormone replacement (Fig. 1). The remarkable findings in the affected individuals in both families are elevated serum thyroglobulin (TG) (Family 1: II-1, 505; Family 2: III-1, III-3, III-4 358, 264, 655, respectively (1.7–55.6 ng/ml) with absent TG antibodies. Additionally, the affected subjects from Family 2 had elevated serum total triiodothyronine (TT3) (258, 228, 245, respectively (76–181 n/dl)). Of note Subject III-2 (Family 3) was on subtherapeutic L-T4 as evidence by TSH of 7.72 (0.4–4.5 mIU/L). Mothers (Family 1: I-2; Family 2: II-2, II-4) had normal thyroid function tests.

**Fig. 2:**
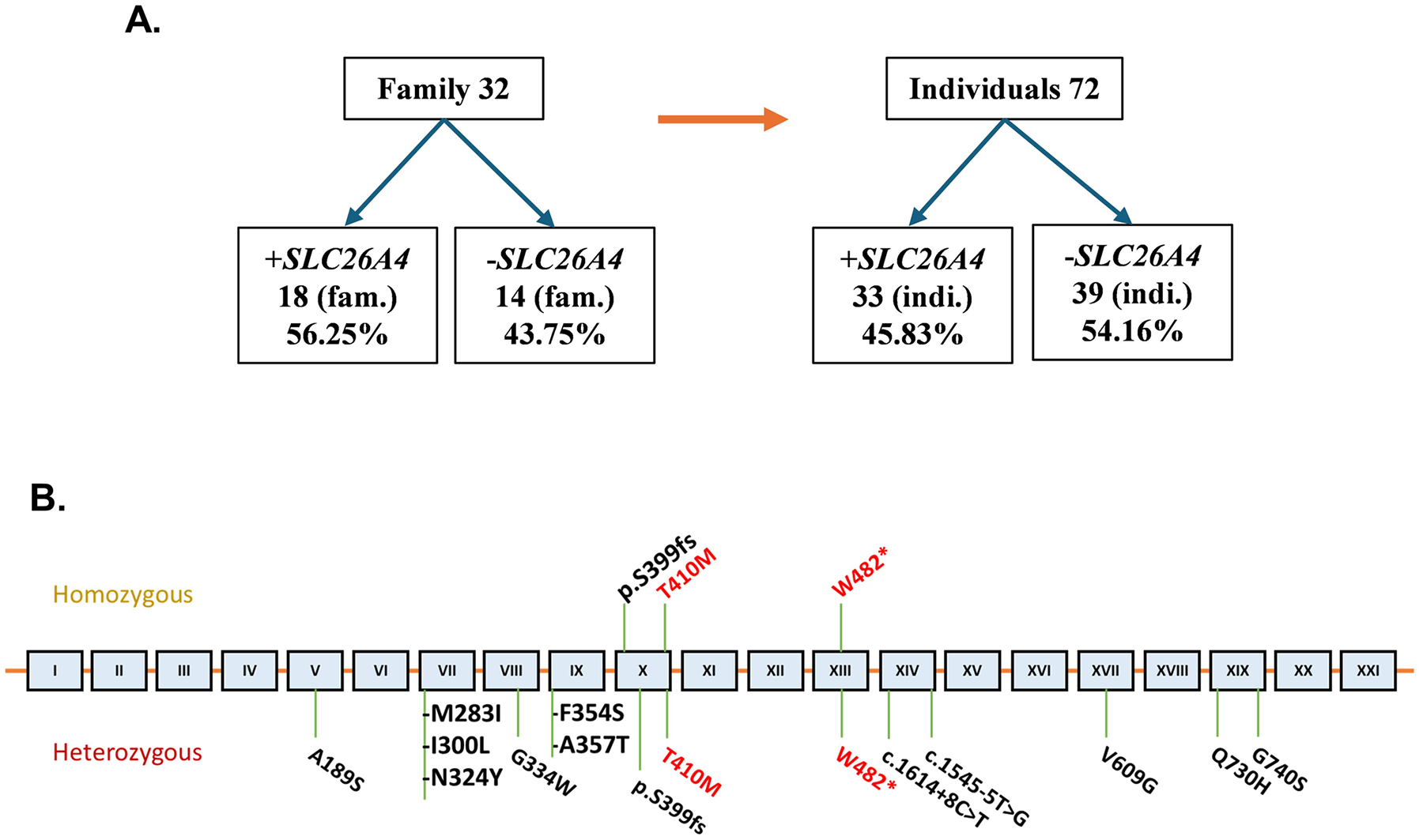
*SCL26A4*variants. **(A)** Prevalence of *SLC26A4* variants in 32 Sudanese families with CH. 56.3% families or, 45.8% individuals harbored *SLC26A4* variants. Of the 33 subjects who tested positive for the variants, 12 (36.4%) harbored more than one *SLC26A4* variant **(B)** Schematic representation of the *SLC26A4* gene and location of variants identified. Amino acid numbers are denoted by numbers spanning schematic. *SLC26A4* exons are denoted by roman numerals. All *SLC26A4* variants identified in the Sudanese individuals in this report are noted at their approximate locations in the *SLC26A4* exons. Variants in red are observed in family 1 and 2

The WES of all affected children revealed abnormalities exclusively involving mutations in the *SLC26A4* gene. In Family 1: II-1 an early homozygous termination variant, c.1446G > A, p.Trp482* in exon 13 was detected. In Family2: III-1, III-3, III-4 a homozygous variant, c.1229C > T, p.Thr410Met in exon 10 was observed (Figs. 1 and 2B). While the predictive capabilities of in-silico algorithms are especially informative for missense variants, the pathogenicity of a nonsense variant such as p.Trp482* is implied by the significant impact of the resulting premature termination on protein function. Thus, based on established ACMG criteria, this variant is classified as ‘likely pathogenic.’ The p.Thr410Met missense variant is predicted to be deleterious based on aggregated results from several in silico tools and is classified as ‘pathogenic’ according to ACMG guidelines (Table 1).

Subsequently Sanger sequencing in both forward and reverse directions confirmed both homozygous variants in the respective probands. The mothers of the probands were heterozygous carriers of the respective variants (Fig. 1).

The *SLC26A4* variants p.Trp482* and p.Thr410Met are either absent or extremely rare in all gnomAD populations but present in our Sudanese cohort, in homozygous states and linked to PDS. These are strong pathogenic variants with potential population-specific enrichment (Table 2).

Additional WES data analysis on DNA from 72 individuals of 32 Sudanese families demonstrated that 56.3% families or 45.8% individuals harbored *SLC26A4* variants either in hetero or homozygous state (Fig. 2A). Of the 33 subjects who tested positive for the variants, 12 (36.4%) harbored more than one *SLC26A4* variant.(Supplemental Table 1).

## Discussion

In Sudan, the absence of routine national newborn screening for CH and hearing have been identified as a significant factor contributing to the late diagnosis of this condition in children [29, 30]. SNHL and goiter in PDS can present at any age. In some cases, the diagnosis is delayed due to the subtlety of symptoms or misinterpretation of clinical signs [31]. Early detection and intervention are key to ensuring that the children have the best possible chance for normal development.

The proband of family 1 who presented with goiter, hypothyroidism and SNHL, was homozygous for *SLC26A4* p.Trp482*, leading to a premature stop codon at amino acid 482 (Fig. 1). All truncation defects identified have been previously found to abolish pendrin function [32, 33]. The p.Trp482* variant was previously reported as part of a compound heterozygous defect together with p.Gly102Arg in a PDS patient with a less severe phenotype and without goiter [34]. Co-segregation analysis for the family revealed these two variants to be compound heterozygous and both were classified as pathogenic. Another PDS patient with goiter and hypothyroidism was due to the p.Gly102Arg homozygous variant [35]. In addition, SNHL without any indications of abnormal thyroid function or EVA was reported with the same variant [36]. A minigene splicing assay of p.Gly102Arg detected abnormally spliced transcripts which are responsible for altered pendrin function and loss of iodide efflux [37, 38].

The proband of family 2 with goiter and hypothyroidism, was homozygous for p.Thr410Met. Hearing loss was variable among the affected members. This variant was previously reported as a part of a compound heterozygous defect together with a p.Val659Leu in a Chinese patient with bilateral profound hearing loss without goiter [39]. p.Val659Leu was also reported in an east Indian subject in homozygous state manifesting with bilateral profound hearing loss but no goiter and normal thyroid function. However, this individual’s affected elder brother was diagnosed with the same variant and had profound hearing loss and goiter [40]. In vitro functional study of this variant demonstrated impaired protein trafficking to the plasma membrane and consequently loss of iodide efflux [40, 41].

That the phenotype of PDS is variable in the same family with the same variant has been previously appreciated [34]. Different levels of disease progression and impairment have been reported in identical twins with PDS [42]. In this later report one sibling had benign thyroid nodules whereas the other twin developed papillary thyroid carcinoma [42]. Furthermore, a wide variety of phenotypes associated with *SLC26A4* variants were shown by inter- and intra-familial levels [34, 40]. Additional genotype-phenotype correlations report compound heterozygous *SLC26A4* variants presenting with high frequency hearing loss and EVA (Mondini malformation) without goiter, manifesting more severe deafness, earlier age of onset, and more variable hearing levels [43–45].

Variants in *SLC26A4* causing PDS can also be di-genic with other genes such as *FOX1*, *EPHA2*, and *KCNJ10* [46–50]. In our study no candidate disease causing variants were detected in these genes. This suggests that the *SLC26A4*-related phenotypes in our families are driven by the *SLC26A4* gene.

The intake of iodine affects the onset of thyroidal abnormalities and the development of goiter in PDS patients [6]. Dietary iodine deficiency in addition to various etiological factors contribute to goiter endemicity in the Sudan [51]. Sudan is recognized as a country with moderate-to-severe iodine deficiency, and this deficiency has been associated with increased thyroid volume and elevated TSH levels when compared with iodine-sufficient regions [6, 52]. In our cohort, several affected children exhibited elevated serum thyroglobulin and goiter in the setting of pathogenic *SLC26A4* variants, findings consistent with the interplay between genetic susceptibility and chronic iodine deficiency [53]. Although we did not perform systematic assessments of thyroid volume or dietary iodine intake, the available thyroid function data (TSH, FT4, and TG) support the view that iodine deficiency may exacerbate thyroid enlargement and dysfunction. These observations underscore the importance of considering local iodine status when interpreting thyroid phenotypes in PDS.

Evaluating the prevalence of the *SLC26A4* gene variants in our cohort of 32 Sudanese families with CH comprising 72 individuals, irrespective of their gender and age, we report that 56.3% families and 45.8% individuals are harboring *SLC26A4* variants, most of them being heterozygous (Table 2; Fig. 2). The analysis highlights that 12 subjects of the 33 positive for the variants (36.4%) harbored more than one *SLC26A4* variants. Current variants p.Trp482* and p.Thr410Met (Fig. 1) along with *SLC26A4* p.Ser399fs (Supplemental Fig. S1), *TPO* and *TG* variants studied previously [1] are disease causing in recessive state. Our data suggest that consanguinity accounts at least in part for high prevalence of CH in Sudan, while the low iodine increases the manifestation of hypothyroidism and goiter being 3 times higher in the Sudanese compared to other populations [1]. Recurrent homozygous variants such as p.Trp482 and p.Thr410Met were observed in our cohort. Although such recurrence might raise the possibility of a founder effect, haplotype analysis was not performed, and therefore a founder mutation cannot be confirmed. An alternative explanation is the high rate of consanguinity and inbreeding within the studied population [54, 55], which increases the likelihood of homozygosity for rare alleles. This demographic factor may therefore contribute to the increased prevalence of these variants in our cohort, underscoring the importance of considering population structure when interpreting recurrent mutations in PDS.

Regarding the functional consequences of the nonsense p.Trp482*, its resulting abnormal mRNA transcript with premature termination is expected to be subject to nonsense-mediated mRNA decay (NMD) [56]. This quality control pathway in cells degrades mRNA molecules containing premature termination codons thus preventing the production of truncated and potentially harmful proteins. From point of view of the putative effect of this variant on the protein, its localization within transmembrane domain 13 results in a loss of almost 300 amino acids compared to the 780 amino acids normal pendrin. Structural modeling using Swiss-Model further suggests significant conformational disruption that could impair protein function (Supplemental Fig. S2). The consequences of the missense variant p.Thr410Met, has been functionally characterized previously as part of a larger genotype phenotype study of patients with bi-allelic *SLC26A4* variants [57].

To our knowledge, this is the first report combining clinical and molecular analysis of PDS in Sudanese patients, although an earlier case was documented without molecular data [58]. We have notified the caring physicians that the affected children require regular follow-ups to maintain adequate thyroid hormone replacement. Audiology testing should be done as early as possible to implement the appropriate treatment. Unfortunately, follow up is severely limited due to ongoing crises in Sudan. The other children who are currently not yet studied should undergo thorough thyroid and audiology evaluations and treatment as indicated.

## Strengths and limitations

This study provides novel insights into *SLC26A4*-related disorders in a Sudanese cohort with congenital hypothyroidism, integrating detailed clinical, biochemical, and molecular analyses. However, the relatively small sample size (32 families; 72 individuals) limits the generalizability of our findings and our ability to draw firm conclusions regarding the population-level prevalence or allelic enrichment of *SLC26A4* variants in Sudan. Future studies with larger, more geographically diverse cohorts will be important to confirm and expand upon these observations. Moreover, although recurrent homozygous variants (e.g., p.Trp482, p.Thr410Met) were observed, we could not confirm a founder effect without haplotype analysis, and their recurrence may instead reflect the high rate of consanguinity in this population.

## Conclusion

PDS may be more common than previously appreciated in the Sudanese population, with consanguinity and chronic iodine deficiency contributing to its expression. Early diagnosis and treatment are essential to prevent the sequalae of hypothyroidism and hearing deficit. Recurrent *SLC26A4* variants observed in our cohort likely reflect high consanguinity rather than a founder effect. These findings support including *SLC26A4* in genetic testing of children with congenital hypothyroidism or early-onset SNHL in this population. The characterization of additional variants will contribute to a better understanding of the phenotypic variability with PDS in Sudan.

## Supplementary Material

supplements Material

Supplem material

Supplem Material

**Supplementary Information** The online version contains supplementary material available at https://doi.org/10.1007/s12020-025-04423-4.

## Figures and Tables

**Table 1:** In Silico predictions of *SLC26A4* variants and ACMG classification

SLC26A4 Variants	in silico Predictions							in silico Aggregated Prediction	ACMG Classification
	SIFT	Poly-phen2	FATH-MM	Mutation Taster	METAL	Mutation Assessor	REVEL		
c.565G > T, p.Ala189Ser	0.13,T	0.03, B	−3.70, U	0.99, D	0.73, D	1.34, L	0.48, B	Uncertain	B (PP2, BS1, BS2, BP6)
c.849G > C, p.Met283Ile	0.44, T	0.28, B	−3.08, U	0.97, D	0.41, U	−0.24, B	0.39, B	Uncertain	B (PP2, BS1, BS2, BP6)
c.898 A > C, p.Ile300Leu	< 0.01, D	1.00, D	−3.16, U	1.00, D	0.69, U	1.58, L	0.69, D	Deleterious	B (PP3, PP2, BS1, BS2, BP6)
c.970 A > T, p.Asn324Tyr	0.01, D	1.00, D	−3.20, U	0.98, D	0.62, U	2.75, M	0.78, D	Deleterious	B (PP3, PP2, BA1, BS2, BP6)
c.1000G > T, p.Gly334Trp	0.00, D	1.00, D	−3.51, U	1.00, D	0.94, D	3.65, H	0.98, D	Deleterious	LP (PP3, PM2, PM5, PP2)
c.1061T > C, p.Phe354Ser	< 0.01, D	1.00, D	−3.15, U	1.00, D	0.87, D	2.49, M	0.95, D	Deleterious	LP (PP3, PM2, PP2, BP6)
c.1069G > A, p.Ala357Thr	< 0.01, D	0.62, P	−3.53, U	1.00, D	0.81, D	1.78, L	0.85, D	Deleterious	B (PP3, PP2, BS1, BP6)
^A^ c.1197delT, p.Ser399fs	-	-	-	-	-	-	-	-	P (PM3, PVS1, PM2, PP5)
^B^ c.C1229T, p.Thr410Met	0.00, D	1.00, D	−3.61, U	1.00, D	0.93, D	3.26, M	0.91, D	Deleterious	P (PP1, PS3, PM1, PP2, PM2, PM5, PP3, PP5)
^C^ c.G1446A, p.Trp482*	-	-	-	-	-	-	-	-	LP (PVS1, PM2)
c. 1545–5T > G	-	-	-	-	-	-	-	-	B (BA1, BS2, BP4, BP6)
c.1614 + 8 C > T	-	-	-	-	-	-	-	-	LB (PM2, BP4, BP6)
c.1826T > G, p.Val609Gly	0.55, T	0.00, B	−3.20, U	0.88, N	0.01, B	−1.06, B	0.27, B	Uncertain	B (PP2, BA1, BS2, BP6)
c.2190G > T, p.Gln730His	0.05, U	0.02, B	−3.30, U	0.91, D	0.63, U	0.90, L	0.46, U	Uncertain	LB (PP2, BS2, BP6)
c.2218G > A, p.Gly740Ser	0.09, T	0.03, B	−3.09, U	< 0.01, N	0.20, U	0.00, B	0.40, U	Uncertain	B (PM5, PP2, BA1, BS2, BP6)

A Previously reported (ref. 1; details in Supplemental Table 1)

B Family 2 in this report

C Family 1 in this report

Sorting Intolerant From Tolerant (SIFT). Scores and predictions are separated by a comma. The score can range from 0 to 1. There are two possible predictions: D (damaging, score ≤ 0.05); T (tolerated, score ≥ 0.05)

Polymorphism Phenotyping (Polyphen2). The score can range from 0 to 1. Three possible predictions: D (probably damaging, score ≥ 0.909), P (possibly damaging, 0.447 ≤ score ≤ 0.908), B (benign, score ≤ 0.446)

Functional Analysis Through Hidden Markov Models (FATH-MM). The score can range from − 16.13 to 10.64. Two possible predictions: D (probably damaging, score <−1.5), U (Uncertain or Benign, score <−1.5)

Mutation Taster. The score can range from 0 to 1. Four possible predictions: A (disease-causing automatic), D (disease-causing), N (polymorphism), P (polymorphism automatic)

Fast and Efficient Meta-Analysis of Genomewide Association Scans (METAL). The score can range from 0 to 1. Higher values are more likely to be deleterious

Mutation Assessor. Score range is −5.135 to 6.49, score < 0.8 Benign (B), (0.8,1.95) Low deleterious probability (L), (1.935, 3.5) Medium deleterious probability (M), > 3.5 High deleterious probability (H)

Rare Exome Varian Ensemble Learner (REVEL). The score for an individual missense variant can range from 0 to 1, with higher scores reflecting the greater likelihood that the variant is disease-causing. Two possible predictions: D (likely disease causing, score > 0.5), B (likely Benign, score < 0.5)

ACMG, American college of medical genetics and genomics; B, benign; LB, likely benign; LP, likely pathogenic; P, pathogenic; PM1, pathogenic moderate (Non-truncating non-synonymous variant is located in a mutational hot spot and/or critical and well-established functional domain); PM2, pathogenic moderate (Extremely low frequency in gnomAD population databases); PM3, pathogenic very strong (For recessive disorders, detected in trans with a pathogenic variant, or in a homozygous or compound heterozygous state in affected cases); PM5, pathogenic Supporting (Different amino acid change as a known pathogenic variant); BA1, benign stand alone (Allele frequency is common for disease in population databases); BS2, benign Strong (Variant was observed in a homozygous state in population databases more than expected for disease); BP4, benign Supporting (For a missense or a splice region variant, computational prediction tools unanimously support a benign effect on the gene); BP6, benign Strong (Reputable source recently reports variant as benign, but the evidence is not available to the laboratory to perform an independent evaluation); BS1, benign strong (Allele frequency is greater than expected for disorder); PP1, pathogenic strong (Cosegregation with disease in multiple affected family members in a gene definitively known to cause the disease); PP2, pathogenic supporting (Missense variant in a gene with low rate of benign missense mutations and for which missense mutation is a common mechanism of a disease); PP3, pathogenic Supporting (For a missense or a splicing region variant, computational prediction tools unanimously support a deleterious effect on the gene); PP5, pathogenic Supporting (Reputable source recently reports variant as pathogenic, but the evidence is not available to the laboratory to perform an independent evaluation); PS3, pathogenic supporting (Well-established functional studies show damaging effect on the gene or gene product), PVS1, pathogenic very strong (Null variant in a gene where loss of function is a known mechanism of disease)

**Table 2:** *SLC26A4* gene variants found in 18 out of 32 Sudanese families with CH

Mutation	Individuals (%)	Clinical PDS	Allele frequency (gnomAD) in African/African American	Allele frequency (gnomAD) East Asian	Allele frequency (gnomAD) European	Allele frequency (gnomAD) Middle Eastern	Total allele frequency (gnomAD)
c.565G > T, p.Ala189Ser	1 (1.39) - Hetero	-	0.01229	0.00000	0.000006781	0.00000	0.0006258
c.849G > C, p.Met283Ile	4 (5.55) - Hetero	-	0.009534	0.00000	0.0001094	0.001191	0.0005808
c.898 A > C, p.Ile300Leu	1 (1.39) - Hetero	-	0.01495	0.00000	0.000006781	0.0004969	0.0007646
c.970 A > T, p.Asn324Tyr	2 (2.77) - Hetero	-	0.03466	0.00000	0.00008221	0.001980	0.001803
c.1000G > T, p.Gly334Trp	2 (2.77) - Hetero	-	0.00000	0.00000	0.000002544	0.00000	0.000001860
c.1061T > C, p.Phe354Ser	3 (4.16) - Hetero	-	0.0009996	0.00000	0.0004297	0.008746	0.0005502
c.1069G > A, p.Ala357Thr	1 (1.39) - Hetero	-	0.004973	0.0004950	0.00004577	0.0004950	0.0003030
^A^ c.1197delT, p.Ser399fs	1 (1.39) – Hetero 1 (1.39) – Homo	+	0.00000	0.00000	0.00001017	0.0001645	0.00001364
^B^ c.C1229T, p.Thr410Met	2 (2.77) – Hetero 3 (4.16) – Homo	+	0.00005336	0.0006687	0.0002935	0.00000	0.0001452
^C^ c.G1446A, p.Trp482*	1 (1.39) – Hetero 1 (1.39) – Homo	+	Variant not available	Variant not available	Variant not available	Variant not available	Variant not available
c. 1545–5T > G	5 (6.94) - Hetero	-	0.02868	0.00000	0.0001108	0.001995	0.001628
c.1614 + 8 C > T	3 (4.16) - Hetero	-	0.001198	0.00000	0.00001395	0.0005022	0.00007288
c.1826T > G, p.Val609Gly	14 (19.44) – Hetero	-	0.1415	0.0001561	0.0004165	0.008584	0.007731
c.2190G > T, p.Gln730His	1 (1.39) - Hetero	-	0.003204	0.00000	0.000007655	0.0009908	0.0001858
c.2218G > A, p.Gly740Ser	3 (4.16) - Hetero	-	0.04966	0.00000	0.0001835	0.003472	0.002727

PDS, Pendred Syndrome; + indicates PDS; - indicates no PDS

A Previously reported (ref. 1; details in Supplemental Table 1)

B Family 2 in this report

C Family 1 in this report

## Data Availability

No datasets were generated or analysed during the current study.
