# Supplementary material for: Prevalence of pendrin defects in sudanese families with congenital hypothyroidism
Source: Endocrine. 2025 Sep 16;90(3):1339–49. doi: 10.1007/s12020-025-04423-4 (PMC12690199; doi:10.1007/s12020-025-04423-4)
Supplement: Supplementary file 1 — Supplementary Material 1 [file 12020_2025_4423_MOESM1_ESM.docx]

**Supplemental Table 1. Individuals harboring more than one SLC26A4 variants without PDS from a cohort of 72 Sudanese subjects**

| **Individuals** | **SLC26A4 Variants** | **Family ID^a^** |
| --- | --- | --- |
| 1 | c.565G>T, p.A189S | B |
|  | c.1826T>G, p.V609G |  |
|  | c.1545-5T>G |  |
| 2 | c.1545-5T>G | B |
|  | c.1826T>G, p.V609G |  |
| 3 | c.1000G>T, p.G334W | P |
|  | c.1826T>G, p.V609G |  |
| 4 | c.1000G>T, p.G334W | P |
|  | c.1826T>G, p.V609G |  |
| 5 | c.1826T>G, p.V609G | J |
|  | c.2218G>A, p.G740S |  |
| 6 | c.1826T>G, p.V609G | J |
|  | c.2218G>A, p.G740S |  |
| 7 | c.1826T>G, p.V609G | New |
|  | c.2218G>A, p.G740S |  |
| 8 | c.1826T>G, p.V609G | New |
|  | c.2190G>T, p.Q730H |  |
| 9 | c.1826T>G, p.V609G | Q |
|  | c.1545-5T>G |  |
| 10 | c.1826T>G, p.V609G | New |
|  | c.1614+8C>T |  |
| 11 | c.1545-5T>G | New |
|  | c.1614+8C>T |  |
|  | c.1826T>G, p.V609G |  |
| 12 | p.I300L c.898A>C | New |
|  | c.1545-5T>G |  |
|  | c.1614+8C>T |  |
|  | c.1826T>G, p.V609G |  |

^a^Previously reported (ref. 1)
