# Supplementary figures and images for: Prevalence of pendrin defects in sudanese families with congenital hypothyroidism
Source: Endocrine. 2025 Sep 16;90(3):1339–49. doi: 10.1007/s12020-025-04423-4 (PMC12690199; doi:10.1007/s12020-025-04423-4)

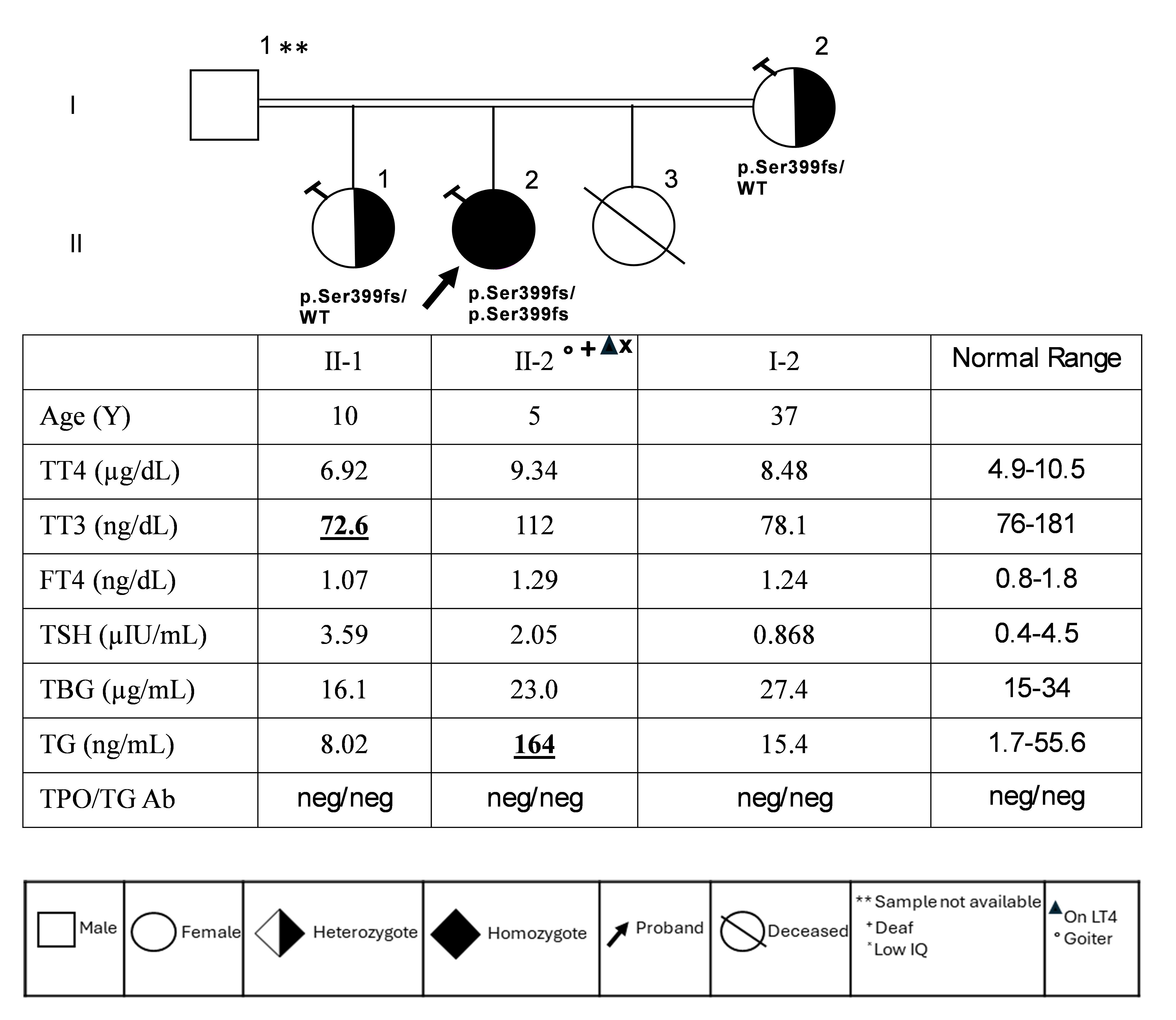

Supplement: Supplementary file 2 — Supplementary Material 2 [file 12020_2025_4423_MOESM2_ESM.jpg]

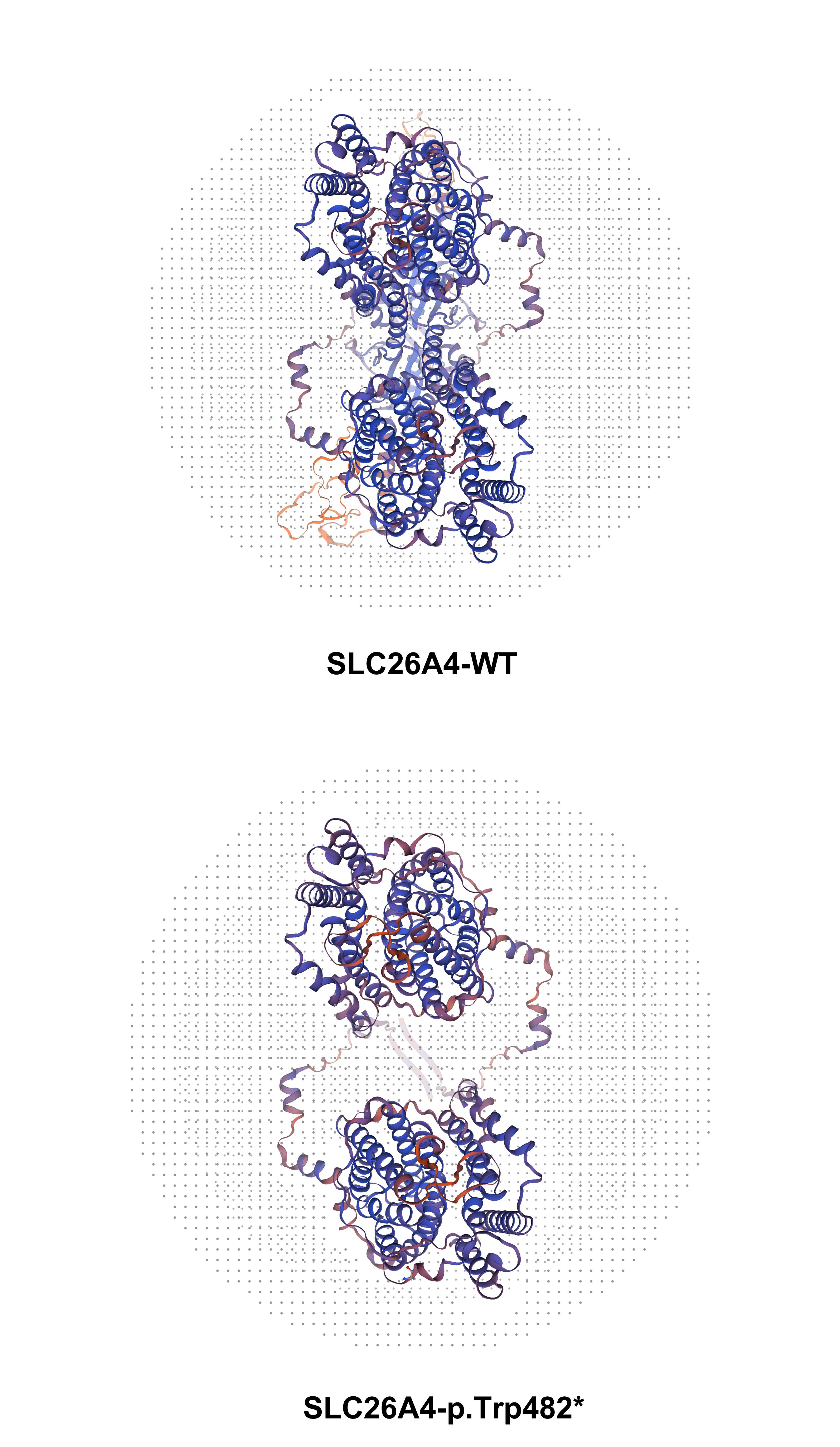

Supplement: Supplementary file 3 — Supplementary Material 3 [file 12020_2025_4423_MOESM3_ESM.jpg]
