# Supplementary material for: Prevalence of pendrin defects in sudanese families with congenital hypothyroidism
Source: Endocrine. Author manuscript; Available in PMC 2025 Dec 11. (PMC12690199; doi:10.1007/s12020-025-04423-4)

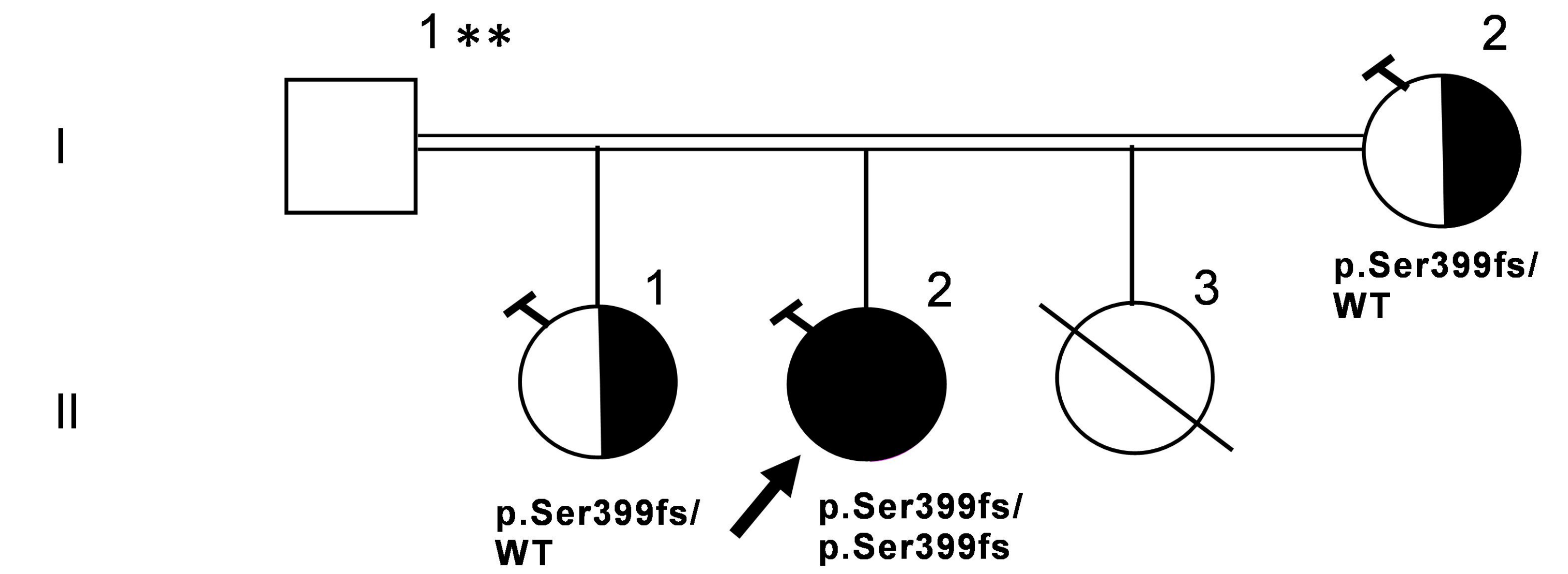

	II-1	II-2 °+▲x	I-2	Normal Range
Age (Y)	10	5	37	
TT4 (μg/dL)	6.92	9.34	8.48	4.9-10.5
TT3 (ng/dL)	**72.6**	112	78.1	76-181
FT4 (ng/dL)	1.07	1.29	1.24	0.8-1.8
TSH (μIU/mL)	3.59	2.05	0.868	0.4-4.5
TBG (μg/mL)	16.1	23.0	27.4	15-34
TG (ng/mL)	8.02	**164**	15.4	1.7-55.6
TPO/TG Ab	neg/neg	neg/neg	neg/neg	neg/neg

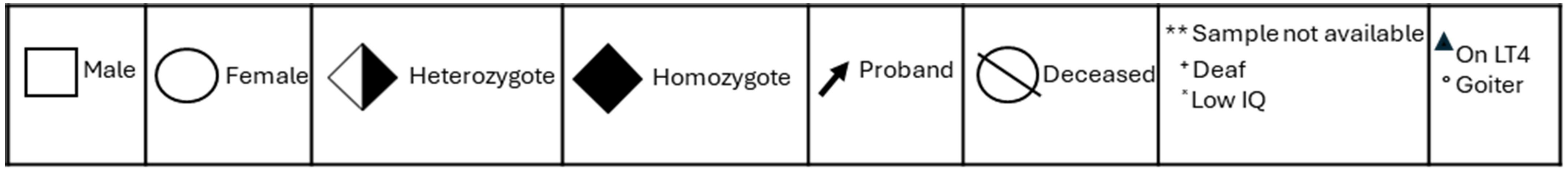

Supplement: Supplementary file 1 [file NIHMS2126980-supplement-Supplem_Material.pdf]
