# Supplementary material for: Prevalence of pendrin defects in sudanese families with congenital hypothyroidism
Source: Endocrine. Author manuscript; Available in PMC 2025 Dec 11. (PMC12690199; doi:10.1007/s12020-025-04423-4)
Supplement: Supplementary file 2 [file NIHMS2126980-supplement-Supplem_material.pdf]

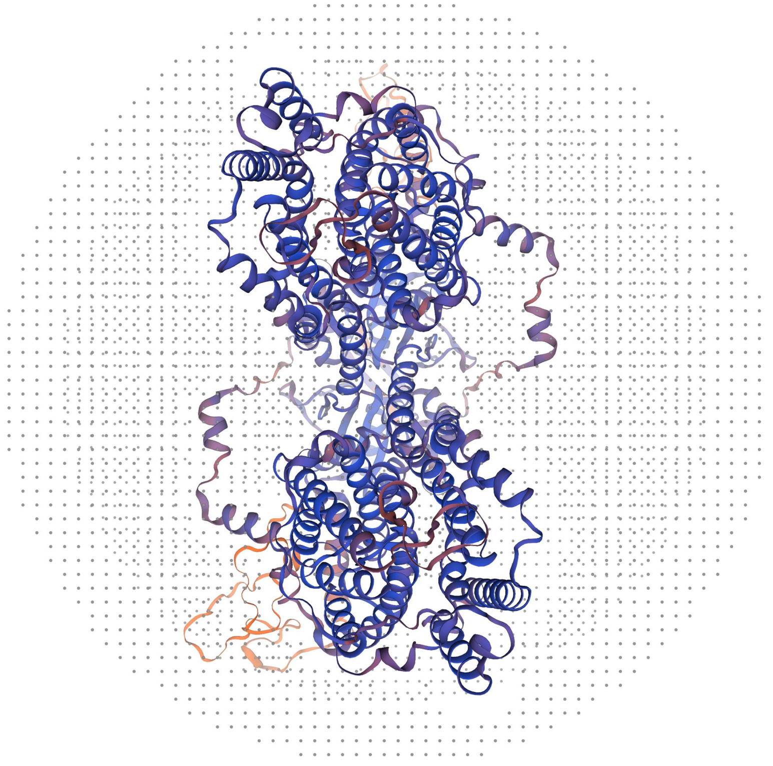

SLC26A4-WT

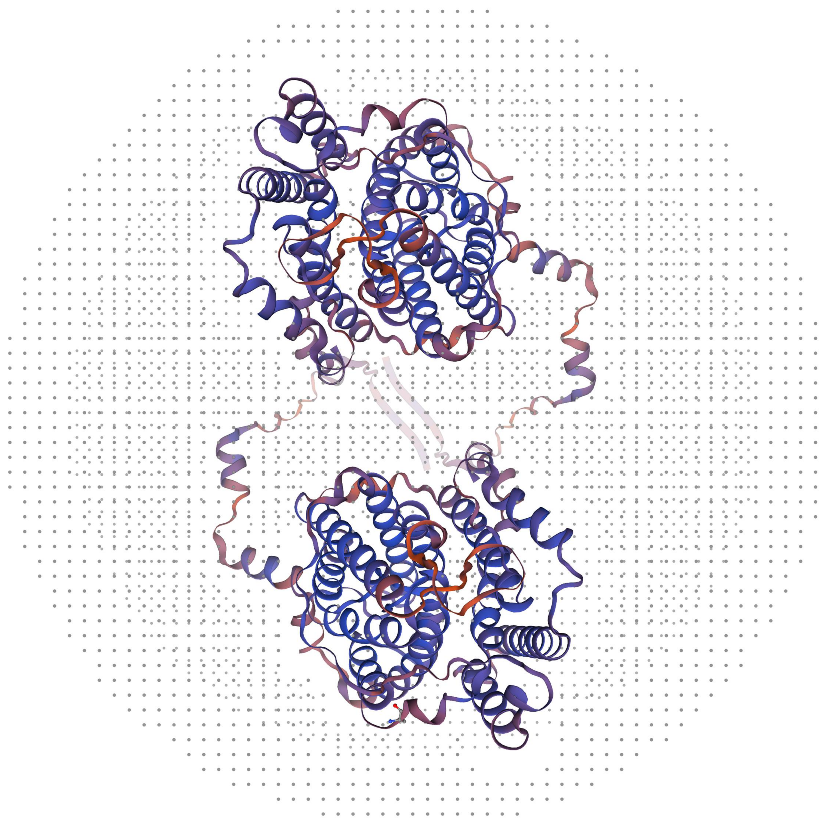

SLC26A4-p.Trp482*
